# Improvements in motor control are associated with improved quality of life following an at-home muscle biofeedback program for chronic stroke

**DOI:** 10.3389/fnhum.2024.1356052

**Published:** 2024-05-15

**Authors:** Octavio Marin-Pardo, Miranda Rennie Donnelly, Coralie S. Phanord, Kira Wong, Sook-Lei Liew

**Affiliations:** ^1^Chan Division of Occupational Science and Occupational Therapy, University of Southern California, Los Angeles, CA, United States; ^2^Stevens Neuroimaging and Neuroinformatics Institute, Keck School of Medicine, University of Southern California, Los Angeles, CA, United States

**Keywords:** biofeedback, quality of life, telerehabilitation, stroke, human-computer interface

## Abstract

**Introduction:**

Chronic stroke survivors with severe arm impairment have limited options for effective rehabilitation. High intensity, repetitive task practice (RTP) is known to improve upper limb function among stroke survivors who have some volitional muscle activation. However, clients without volitional movement of their arm are ineligible for RTP-based interventions and require hands-on facilitation from a clinician or robotic therapy to simulate task practice. Such approaches can be expensive, burdensome, and have marginal effects. Alternatively, supervised at-home telerehabilitation using muscle biofeedback may provide a more accessible, affordable, and effective rehabilitation option for stroke survivors with severe arm impairment, and could potentially help people with severe stroke regain enough volitional activation to be eligible for RTP-types of therapies. Feedback of muscle activity via electromyography (EMG) has been previously used with clients who have minimal or no movement to improve functional performance. Specifically, training to reduce unintended co-contractions of the impaired hand using EMG biofeedback may modestly improve motor control in people with limited movement. Importantly, these modest and covert functional changes may influence the perceived impact of stroke-related disability in daily life. In this manuscript, we examine whether physical changes following use of a portable EMG biofeedback system (Tele-REINVENT) for severe upper limb hemiparesis also relate to perceived quality of life improvements. Secondarily, we examined the effects of Tele-REINVENT, which uses EMG to quantify antagonistic muscle activity during movement attempt trials and transform individuated action into computer game control, on several different domains of stroke recovery.

**Methods:**

For this pilot study, nine stroke survivors (age = 37-73 years) with chronic impairment (Fugl-Meyer = 14-40/66) completed 30 1-hour sessions of home-based training, consisting of six weeks of gaming that reinforced wrist extensor muscle activity while attenuating coactivation of flexor muscles. To assess motor control and performance, we measured changes in active wrist ranges of motion, the Fugl-Meyer Assessment, and Action Research Arm Test. We also collected an EMG-based test of muscle control to examine more subtle changes. To examine changes in perceived quality of life, we utilized the Stroke Impact Scale along with participant feedback.

**Results:**

Results from our pilot data suggest that 30 sessions of remote training can induce modest changes on clinical and functional assessments, showing a statistically significant improvement of active wrist ranges of motion at the group level, changes that could allow some people with severe stroke to be eligible for other therapeutic approaches, such as RTP. Additionally, changes in motor control were correlated with the perceived impact of stroke on participation and impairment after training. We also report changes in corticomuscular coherence, which showed a laterality change from the ipsilesional motor cortex towards the contralesional hemisphere during wrist extension attempts. Finally, all participants showed high adherence to the protocol and reported enjoying using the system.

**Conclusion:**

Overall, Tele-REINVENT represents a promising telerehabilitation intervention that might improve sensorimotor outcomes in severe chronic stroke, and that improving sensorimotor abilities even modestly may improve quality of life. We propose that Tele-REINVENT may be used as a precursor to help participants gain enough active movement to participate other occupational therapy interventions, such as RTP. Future work is needed to examine if home-based telerehabilitation to provide feedback of individuated muscle activity could increase meaningful rehabilitation accessibility and outcomes for underserved populations.

## 1 Introduction

Despite recent advances in stroke rehabilitation, stroke survivors with upper extremity hemiparesis rarely experience full recovery of arm movement control, affecting their participation and independence in daily activities (Winstein et al., 2016). This is in part due to a lack of effective, empirically supported interventions for restoring function in the chronic phase (>6 months post stroke), a gap that is further widened among those with severe impairment (Teasell et al., 2018; Richards and Cramer, 2021). Additionally, chronic stroke survivors with severe impairment in the United States (and other countries) often face healthcare policy barriers that make therapy expensive and inaccessible. For example, shifts from fee-for-service to alternative payment models may result in less therapy for clients with more severe impairment and chronic, complex needs (Brown et al., 2022). Therefore, access is a practical concern affecting the development of interventions for clients with severe, chronic hemiparesis post stroke. Interventions that can be used effectively at home, without synchronous assistance from a therapist, may be a cost-effective and accessible service delivery approach for this underserved population to make progress in their recovery.

High-intensity, repetitive task practice (RTP; also referred to as task-specific training) is a gold standard in physical and occupational therapy for improving post-stroke sensorimotor outcomes. RTP is based on evidence that repetitively practicing specific, goal-oriented movements can induce learning and lead to improved functional performance of those tasks (Winstein et al., 2016). RTP is an important component of current neurorehabilitation approaches and has been shown to be effective in improving arm function (Hubbard et al., 2009; French et al., 2016), both when used in the clinic and in clients’ homes via telerehabilitation (Hsieh et al., 2018). However, a pre-condition for use of RTP-based interventions is having some volitional arm movement (French et al., 2016; Thieme et al., 2018), such as a minimum of 10 degrees of active wrist extension for constraint induced movement therapy (CIMT; Winstein et al., 2003) or a minimum Fugl-Meyer score of 10 for robot-assisted RTP (Dimyan et al., 2022). In these studies, participants who exceeded the minimum requirements had better outcomes than those who narrowly met the eligibility criteria (Wolf et al., 2008; Dimyan et al., 2022). Clients who do not meet these eligibility thresholds for volitional movement can passively simulate task practice with maximum assistance, from robotic therapy (Winstein et al., 2016; Bertani et al., 2017) or hands-on facilitation from a clinician or caregiver, though these approaches are expensive, burdensome, and have marginal effects.

Alternatively, muscle biofeedback approaches, which require only trace muscle activity, may be more accessible and effective rehabilitation options for stroke survivors without volitional movement to potentially help them regain enough volitional activation to be eligible for RTP-based therapies. Biofeedback is used widely in rehabilitation to adjust client’s task performance by transforming biological signals into real-time, extrinsic cues (Giggins et al., 2013). Specifically, electromyography (EMG) biofeedback translates trace muscle activity into sensorimotor feedback (e.g., using the affected arm to control computer games or showing unimpaired movements of a virtual avatar arm). With repeated use, stroke survivors can learn to control their own muscle activity, strengthen functional brain-muscle connections, and regain some volitional movement.

EMG biofeedback has been previously implemented with clients who have moderate to severe post-stroke movement impairment to improve functional performance (Armagan et al., 2003; Lirio-Romero et al., 2021). Specifically, previous work suggests that training to reduce unintended co-contractions of the impaired hand using EMG biofeedback may modestly improve motor control in people with limited movement (Donoso Brown et al., 2014; Marin-Pardo et al., 2020). Importantly, it is unknown whether modest changes in motor control could have impacts on perceived stroke-related disability in daily life or be enough to enable participants to move on to the next “step” of rehabilitation care by making them eligible for other treatments, such as RTP.

In this manuscript, we present pilot data of a portable EMG biofeedback system (Tele-REINVENT) for severe upper extremity hemiparesis. Tele-REINVENT uses EMG to quantify antagonistic muscle activity during movement attempt trials and transform individuated action into computer game control. We hypothesize that at-home training with Tele-REINVENT for 6 weeks will modestly improve scores on clinical measures of motor control and that these changes will be correlated with perceived impact of stroke on daily activities, suggesting wider impacts of subtle motor improvements. In addition, we aim to explore whether participants with different levels of impairment (e.g., moderate versus severe) show distinct improvements.

## 2 Methods

### 2.1 Participants

We recruited nine stroke survivors in the chronic stage of recovery (>6 months after onset). Eligibility criteria included that participants must present with moderate to severe upper extremity hemiparesis and residual hand function (e.g., less than 25 degrees of active wrist extension; trace muscle activity detectable by electromyography). Additional inclusion criteria required that participants were not taking anti-spasticity medication, had no hand contractures, receptive aphasia, a secondary neurological disease, significant vision loss, or severe cognitive impairment. None of the participants received additional physical or occupational therapy targeting wrist movements during the study, but regular exercise was allowed. The experimental protocol was approved by the Institutional Review Board of the University of Southern California, and all participants provided written informed consent in accordance with the Declaration of Helsinki. Table 1 presents a summary of the participant demographics.

**Table 1:** Participant demographics and baseline evaluations.

Characteristic	Baseline measurement
Sex	Males (*n* = 9)
Age (years)	52.78 ± 10.5
Onset (months)	71.33 ± 65.49
Paresis	Left = 6, Right = 3
FMA	26 ± 9.89
ARAT	12.89 ± 8.78
ROM Wrist Extension (°)	8.56 ± 10
MRS	1.88 ± 0.33
MOCA	22.22 ± 1.86

Mean values ± standard deviations for measurements at enrollment. Fugl–Meyer Assessment of the upper extremity (FMA), Range of Motion (ROM), Modified Rankin Scale (MRS), Montreal Cognitive Assessment (MOCA).

### 2.2 Study protocol

We evaluated the functional and perceived outcomes of using Tele-REINVENT for 30 at-home training sessions over 6 weeks. Potential participants were screened for cognitive impairment using the Montreal Cognitive Assessment (MOCA) (Nasreddine et al., 2005) and for muscle activity with a test of EMG amplitude during an in-person visit prior to enrollment. Enrolled participants could sustain a minimum level of wrist extensor EMG activity (i.e., hold 30% of a prerecorded maximum for 10 4-s trials) and did not present a significant cognitive impairment (i.e., having a MOCA score below 20 points).

**Figure 1:**
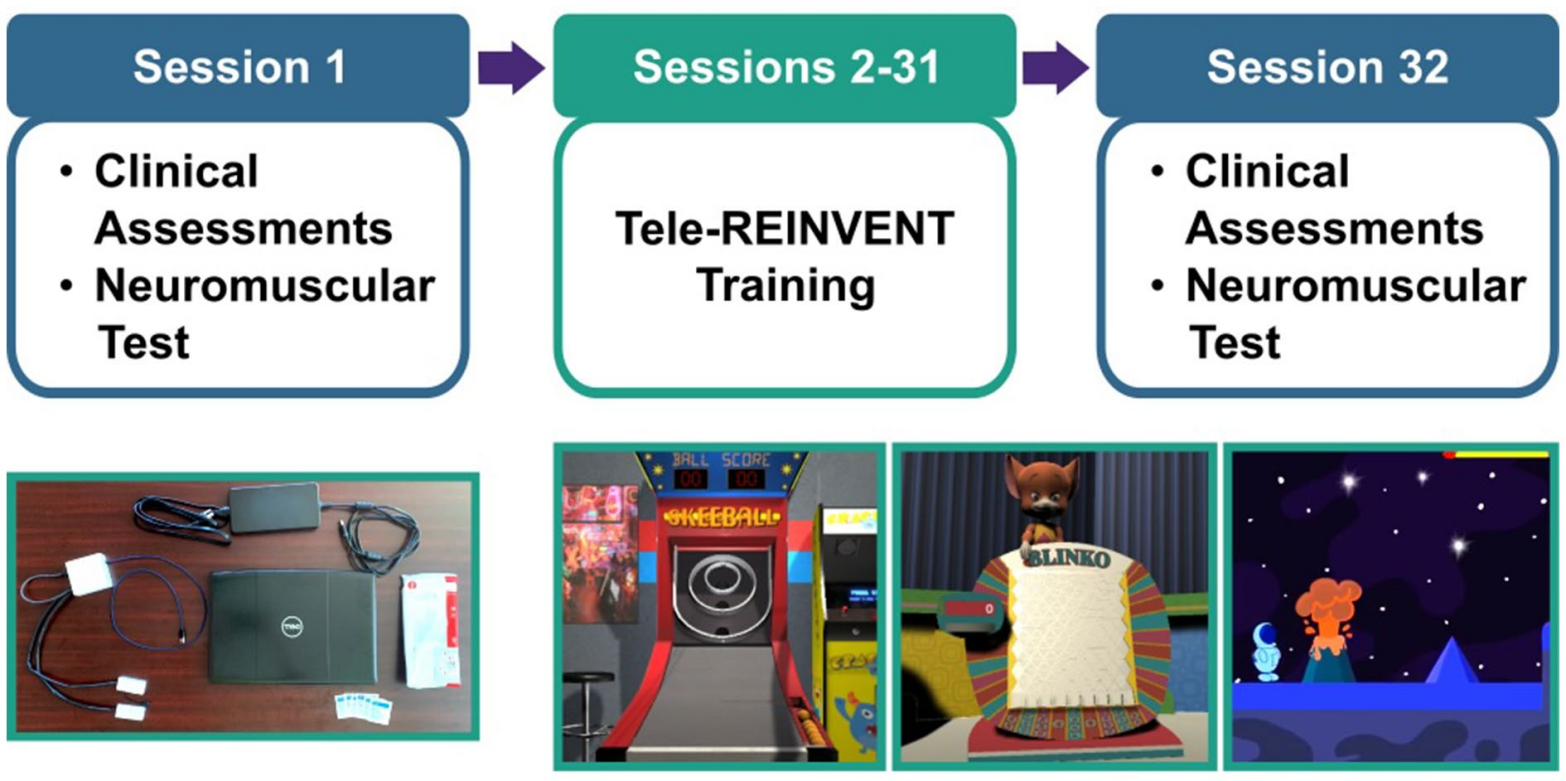
Experimental protocol. (Top) Participants were asked to complete 30 remote sessions (sessions 2–31) targeting the training of wrist movements of the more affected arm. We included a battery of standard clinical assessments and a test of neuromuscular control (i.e., repeated holds of wrist muscle contraction with simultaneous electroencephalography and electromyography recordings) as pre- and post-intervention assessments in sessions 1 and 32. (Bottom, left) Tele-REINVENT system. (Bottom, right) Screenshots of three Tele-REINVENT training games. These games were developed to actively encourage wrist extension movements by providing feedback of individuated muscle activation patterns (e.g., avoiding unintended coactivation of antagonistic muscles).

Our training program is detailed in Marin-Pardo et al. (2022) and the development of the Tele-REINVENT system in Marin-Pardo et al. (2021). Briefly, Tele-REINVENT consists of a laptop computer, a pair of EMG sensors, and a package with disposable electrodes and alcohol wipes (Figure 1). EMG signals are processed in real-time with custom scripts in Matlab (R2021a, The Mathworks, Natick, United States) to quantify a ratio of extensor and flexor muscle activity. This calculation is then used to reward individuated muscle activation via three custom games (Figure 1) presented on the laptop screen:

- *Skeeball*, where individuated extension activity is mapped to score values.
- *Blinko*, where extension and flexion attempts move a character holding a disk across the top of a vertical game board.
- *Planet Jump*, where muscle activity corresponding to extension and flexion movement attempts are translated as jumping and stopping commands.

We evaluated functional improvements with clinical and physiological assessments during pre- and post-training in-person visits to our laboratory in sessions 1 and 32. Our primary clinical endpoints were motor control as measured by wrist ranges of motion (ROM) during active extension and flexion of the impaired hand, perceived impact on quality of life as measured by the Stroke Impact Scale (SIS), and a neurophysiological assessment of muscle control using EMG. We also collected measures of corticomuscular coherence, measuring EMG during wrist extension and flexion while we simultaneously recorded electroencephalography (EEG) signals over the ipsilesional and contralesional brain motor cortices. Finally, we also collected clinical assessments including the Fugl-Meyer Assessment of the upper extremity (FMA) and the Action Research Arm Test (ARAT).

Then, in sessions 2–31, participants were asked to complete an hour of at-home training of individuated wrist extension and flexion EMG feedback. The first week was monitored daily by the research team, whereas subsequent weeks were hybrid (e.g., two monitored sessions and four independent sessions per week). In each training session, participants completed an automated video-guided calibration and then selected which game they would play. We asked them to play their preferred combination of games for at least 1 h per session for a total of 30 sessions. Finally, we obtained participant experience feedback with a questionnaire at the end of each session. This questionnaire included two 10-point Likert rating scales (enjoyment and system function), where 1 corresponded to worse ratings (e.g., *I did not enjoy the session, The system did not work properly*, respectively) and 10 to better ratings (e.g., *I enjoyed the session very much, The system worked without any issues*, respectively). Figure 1 summarizes this training protocol.

### 2.3 Characterization of muscle control via EMG amplitude tracking and corticomuscular coherence

In line with our previous work (Marin-Pardo et al., 2020, 2022), we quantified motor control with a test of muscle contraction where we asked participants to perform 12 trials of quasi-isometric wrist extension with their affected hand. Briefly, in each trial, we used rectified EMG signals to control the movement of a cursor on a computer screen and asked participants to match a target level (15% of a maximal gross grasp) for 4 sec followed by 6 sec of rest. Then, we repeated this assessment during quasi-isometric wrist flexion. For each task, online feedback was provided for the muscle with the largest signal-to-noise ratio during voluntary activation, and practice trials were performed to ensure stable performance. EMG sensors were placed on the extensor carpi radialis longus, extensor carpi ulnaris, flexor carpi radialis, and flexor carpi ulnaris, and signals were acquired at 2148 Hz with a Trigno Wireless system (Delsys Incorporated, Natick, United States). Additionally, we concurrently recorded EEG at 500 Hz with a LiveAmp system (Brain Vision LLC, Morrisville, USA). Electrodes were positioned over the left and right motor cortices using a subset of the 10–10 convention (e.g., FC1, FC5, C3, CP1, CP5, C4, FC2, FC6, C4, CP2, and CP6). All signals were synchronized and recorded using the Labstreaming Layer protocol and recorder (Swartz Center for Computational Neuroscience, 2022) and interpolated to 1,000 Hz for offline analysis.

To quantify muscle individuation, we averaged the last 3 sec of each sustained contraction and calculated the proportion of activity from the extensor muscles to the total recorded activity using Equation 1:


(1) $$ER=\frac{EMG_{\text{extensor}}}{EMG_{\text{extensor}}+EMG_{\text{flexor}}}$$

where ER values closer to 1 would be indicative of extensor muscles being more active than the flexor muscles. By contrast, values closer to 0 would indicate more flexion activity, and values around 0.5 would indicate comparable recruitment from both muscle groups. EMG signals were filtered using a 15–450 Hz bandpass filter, rectified, and normalized to the recorded maximum grasp.

To assess the correlation of brain and muscle activity, we calculated corticomuscular coherence (CMC, a frequency-domain correlation of the two signals) within the beta frequency band (e.g., 12–30 Hz), as this has been suggested to probe corticospinal communication and stroke recovery (Krauth et al., 2019; Liu et al., 2019). We evaluated CMC between the pair of muscles involved in each task (i.e., wrist flexors or wrist extensors) and the ipsilesional and contralesional hemispheres, as previous research has shown bilateral changes in CMC after stroke (Zheng et al., 2018; Krauth et al., 2019; Maura et al., 2023). First, EEG signals were bandpass filtered between 5 and 100 Hz, re-referenced to the common average after removing noisy or bad channels using an EEGLAB artifact detection method (Delorme and Makeig, 2004; Kothe and Jung, 2016), and z-scored. Then, we used channels C3 and C4 to calculate their coherence with Hilbert-transformed and z-scored EMG signals using 128 ms Hann-windowed segments with 75% overlap. Finally, we calculated the 95% confidence level for each profile [i.e., 1–0.05^(1/(L-1)], where L corresponds to the adjusted number of segments used) (Kattla and Lowery, 2010). Since previous studies have shown that CMC is not precisely localized in this study population (Rossiter et al., 2013; Krauth et al., 2019; Marin-Pardo et al., 2020), if C3 or C4 channels were removed, we used a neighboring electrode corresponding to the respective sensorimotor hemisphere.

### 2.4 Statistical analyses

Offline signal processing and statistical analyses were performed using custom scripts in Matlab (R2022b, The Mathworks, Natick, United States) and R (4.3.2, R Foundation for Statistical Computing, Vienna, Austria), respectively.

#### 2.4.1 Changes in motor control

We used paired t-tests to identify consistent changes in wrist ROM, SIS, and muscle individuation (via EMG) across the group. For muscle individuation, we used Equation 1 to calculate ratios of muscle-group activity and quantify the involvement of extensor and flexor muscles while performing the extension and flexion EMG tracking tasks. Then, we calculated mean ER values for each trial and evaluated group-level effects with participants’ mean values for each task. As these assessments quantify different domains of motor control (e.g., function, quality of life, muscle co-activation), we considered these group-level tests as independent and thus significant at *p* < 0.05.

Then, we explored possible relationships between quantitative (e.g., change in muscle individuation during wrist extension and ROM) and perceived motor improvements (e.g., SIS subscales). As current stroke rehabilitation guidelines and models encourage focusing on client’s performance and participation (Heerkens et al., 2018; Hildebrand et al., 2023), we sought to evaluate these domains, aligning with the International Classification of Functioning, Disability and Health (World Health Organization, 2001), using corresponding subscales on the SIS. Specifically, we calculated Impairment as the normalized score of the Strength subscale, Function as the combined scores of the normalized ADL and Hand Function subscales, and Participation as the normalized Participation subscale score. Then, we used matrix correlations to investigate these associations with muscle individuation and wrist ROM, separately. Correlations were considered significant at *p* < 0.05 (controlling for a false discovery rate using the Benjamini and Hochberg method).

#### 2.4.2 Neuromuscular changes following training

We evaluated laterality changes (i.e., which brain hemisphere had increased coherence, relative to the overall coherence of both hemispheres) to investigate changes in neural reorganization. We calculated beta band CMC at the group level for each task (flexion and extension) using Equation 2:


(2) $$\text{Laterality}=\frac{Coh_{\text{ipsilesional}}-Coh_{\text{contralesional}}}{Coh_{\text{ipsilesional}}+Coh_{\text{contralesional}}}$$

where Coh represents the Fisher-transformed coherence (i.e., atanh (sqrt (coherence)) for each hemisphere (ipsilesional and contralesional) and muscle group (extensors and flexors) averaged across the beta band using Stouffer’s method. We then used paired t-tests to assess mean changes for each task with a significance of *p* < 0.05.

Additionally, we explored associations between changes in ipsilesional peak CMC within the beta band during flexion and extension movements and changes in FMA with Pearson correlations, with a significance threshold of *p* < 0.05.

#### 2.4.3 Behavioral changes In clinical assessments

We used paired t-tests to identify consistent changes in FMA and ARAT across the group, setting the threshold for significance at *p* < 0.05.

## 3 Results

In this study we aimed to evaluate the relationship between functional and perceived outcomes of using Tele-REINVENT with nine moderately and severely impaired stroke survivors in the chronic stage of recovery (>6 months after onset).

### 3.1 Changes in motor control

**Figure 2:**
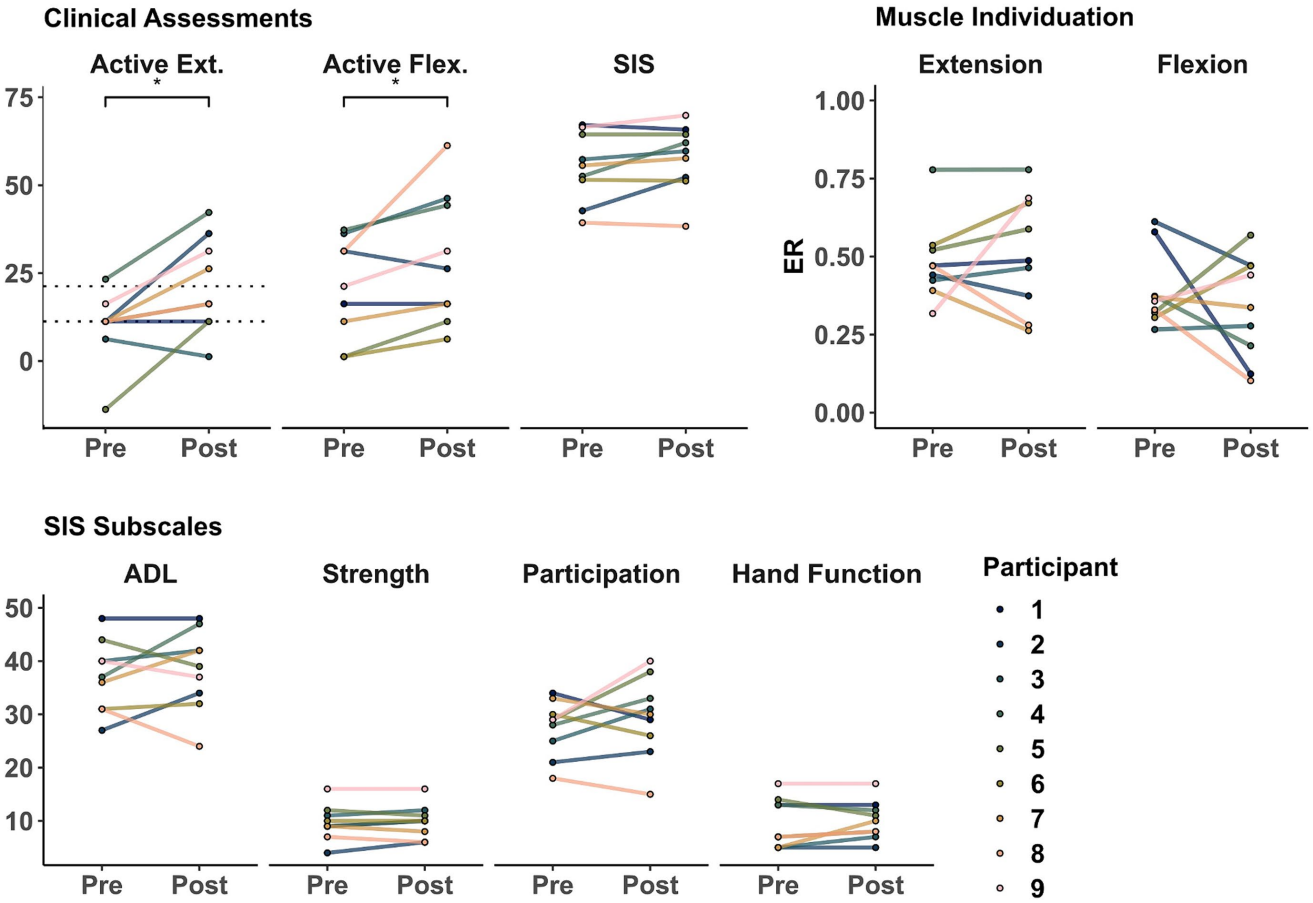
Clinical and physiological assessments before and after training. Markers represent the score for each participant as evaluated in sessions 1 (Pre) and 32 (Post). (Top Left) Active range of motion during wrist extension and flexion and Stroke Impact Scale (SIS). (Top Right) muscle individuation (ER) during wrist extension and flexion. (Bottom) SIS upper extremity motor-related subscales. Dotted lines represent 10 and 20 degrees of active wrist extension, showing lower- and higher-functioning groups, respectively (as defined in Winstein et al. (2003); Wolf et al. (2010)). *indicate a significant difference of *p* < 0.05.

There were group-level improvements in motor control across different domains of recovery. As seen in Figure 2 and Table 2, there were significant improvements at the group level in active wrist extension (*t* = 3.20, *p* = 0.012) and flexion (*t* = 2.48, *p* = 0.038). However, these changes were not significant for ER during extension (*t* = 0.50, *p* = 0.630), ER during flexion (*t* = −0.78, *p* = 0.456), or SIS (*t* = 1.92, *p* = 0.091) at the group level. Additionally, a summary of the SIS subscales that relate to upper extremity motor impairment (e.g., Participation, Activities of Daily Living (ADL), overall Strength, and Hand Function) is presented in Table 3. There were no significant changes at the group level in these SIS subscales.

**Table 2:** Behavioral assessments before and after training.

Assessment	*t*	*p*	Pre	Post
Active extension	**3.20**	0.012*	**8.56 ± 10**	**20.11 ± 13.42**
Active flexion	**2.48**	0.038*	**19.56 ± 14.16**	**27.56 ± 18.51**
ER (extension)	0.50	0.630	0.48 ± 0.13	0.51 ± 0.18
ER (flexion)	−0.78	0.456	0.39 ± 0.12	0.33 ± 0.17
SIS	1.92	0.091	54.88 ± 9.92	57.56 ± 9.54

Mean values ± standard deviations included for pre- and post-intervention evaluations. Muscle individuation quantified with an extension ratio (ER) during wrist extension and flexion movement, Stroke Impact Scale (SIS). *and bold fonts indicate significance of *p* < 0.05.

**Table 3:** Motor-related stroke impact scale subscales before and after training.

SIS Subscale	*t*	*p*	Pre	Post
ADL	0.64	0.538	37.11 ± 6.72	38.33 ± 7.60
Hand Function	0.76	0.468	9.56 ± 4.67	10.11 ± 3.62
Participation	0.99	0.348	27.44 ± 5.27	29.44 ± 7.60
Strength	0.61	0.559	9.22 ± 2.54	9.44 ± 2.97

Mean values ± standard deviations included for pre- and post-intervention Stroke Impact Scale (SIS) subscales. Activities of Daily Living (ADL).

### 3.2 Correlation between objective motor control and subjective quality of life outcomes

**Figure 3:**
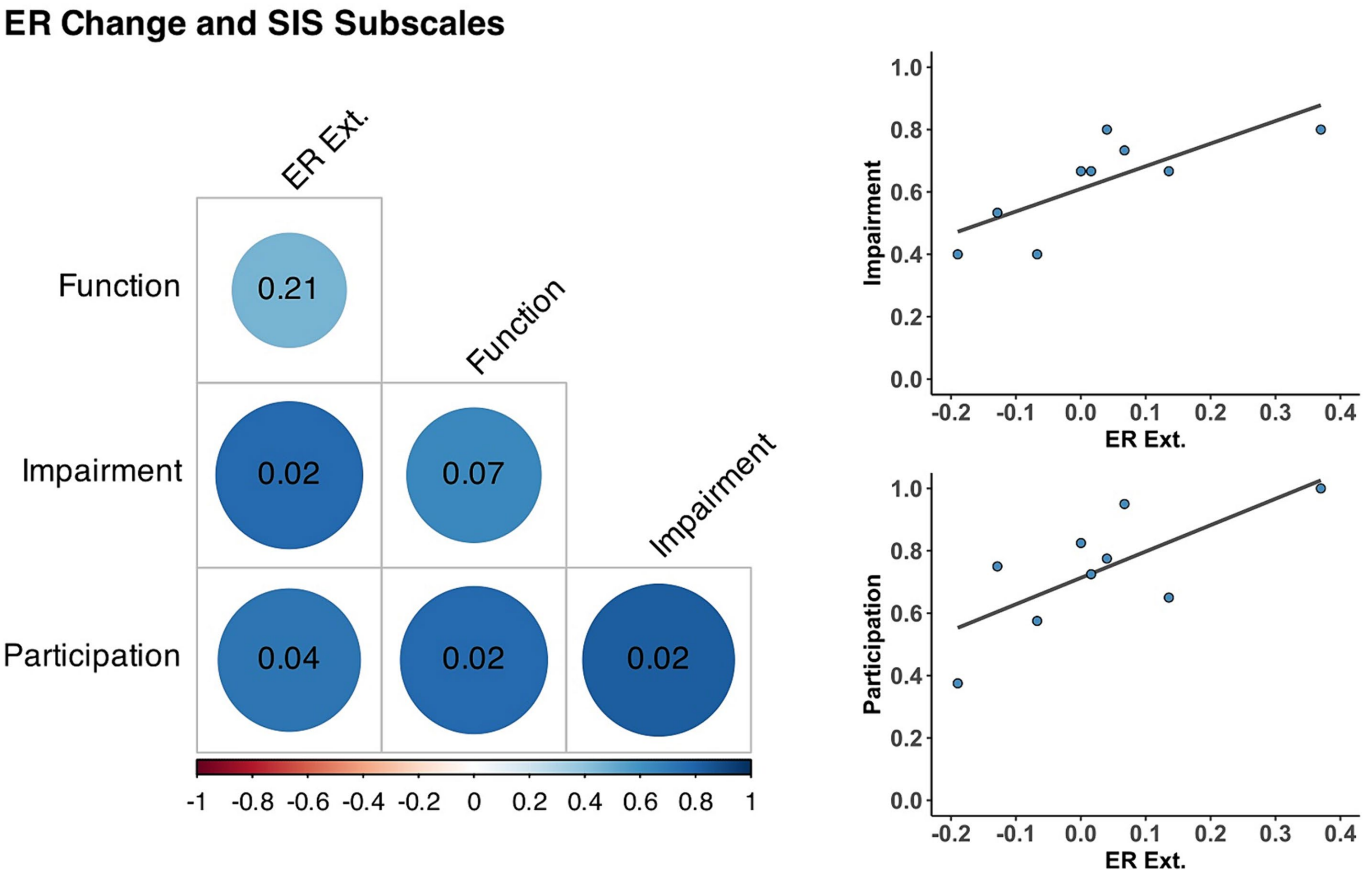
Correlations between muscle individuation and Stroke Impact Scale Subscales. (Left) Correlation matrix between individuated wrist extension changes (ER ext.) and Stroke Impact Scale subscales after training. Blue circles represent positive correlations at the intersection of each row and column, with their respective *p*-value (corrected for multiple comparisons) inside the cell and darker circles indicate stronger correlations. (Top Right) Correlation between ER ext. and Impairment (*r* = 0.77, *p* = 0.024). (Bottom Right) Correlation between ER ext. and Participation (*r* = 0.72, *p* = 0.036).

Importantly, participants showed a positive correlation between improvement of individuated wrist extension and post-intervention SIS. As shown in Figure 3 and Table 4, there was a significant positive correlation between ER improvement during wrist extension and the SIS measures of Participation (*r* = 0.72, *p* = 0.036) and Impairment after the intervention (*r* = 0.77, *p* = 0.024) but not with Function (*r* = 0.46, *p* = 0.208). Extension ER was not significantly correlated with Participation (*r* = 0.37, *p* = 0.326), Impairment (*r* = 0.66, *p* = 0.104), or Function (*r* = 0.57, *p* = 0.151) before training. Active extension ROM was not correlated with SIS subscales before or after the intervention. For completeness, we also explored correlations with ER change and ROM during active flexion. However, quality of life outcomes were not significantly correlated with these assessments.

**Table 4:** Correlations between changes in muscle individuation during wrist extension and post-intervention perceived impact of stroke.

	Pre		Post	
Correlation	*r*	*p*	*r*	*p*
ER extension – SIS Function	0.57	0.151	0.46	0.208
ER extension – SIS Participation	0.37	0.326	0.72	0.036*
ER extension – SIS Impairment	0.66	0.104	0.77	0.024*

SIS, Stroke impact scale. *and bold fonts indicate significance of *p* < 0.05 (corrected for multiple comparisons).

### 3.3 Neuromuscular changes after training

**Figure 4:**
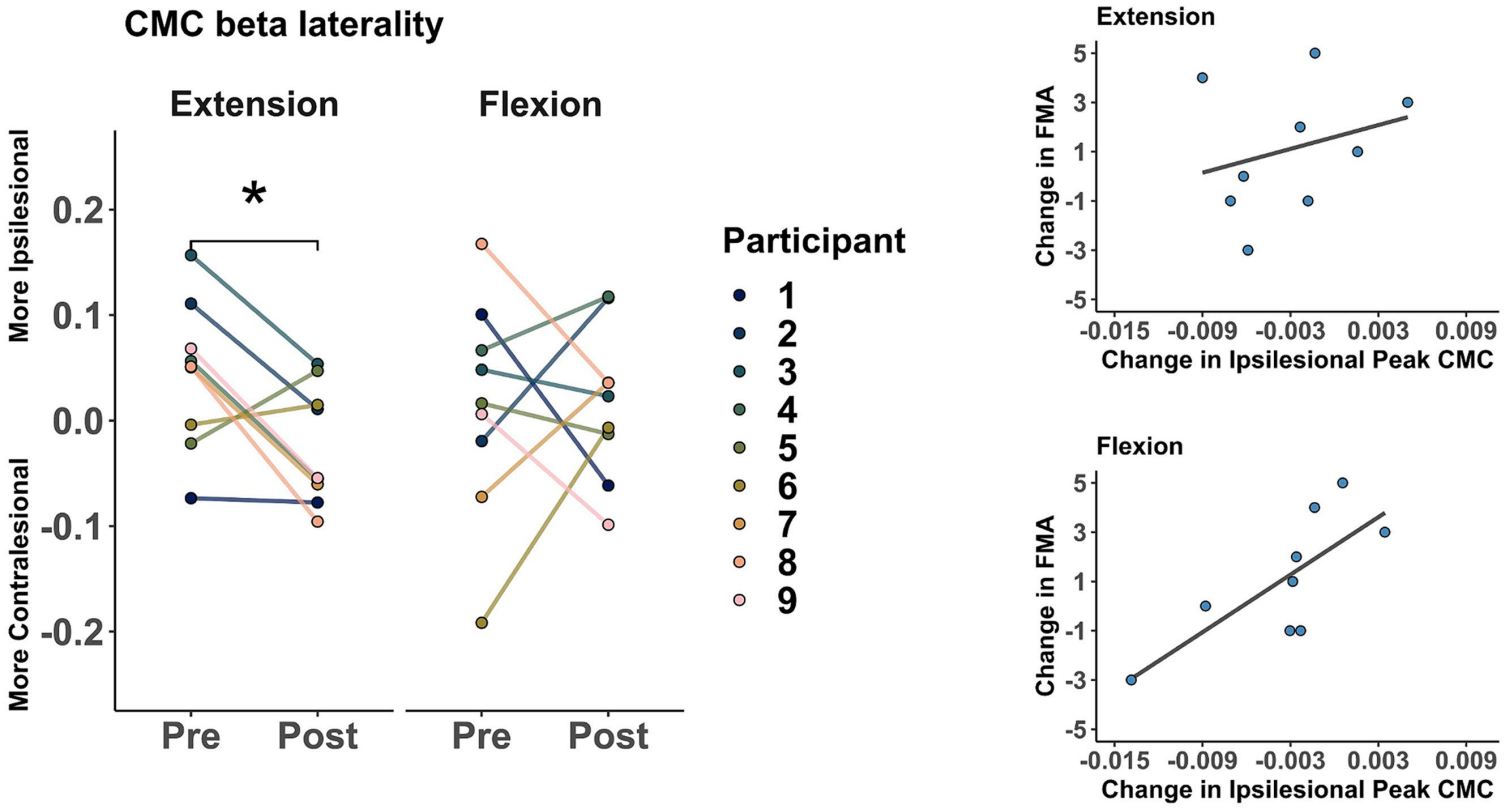
Changes in corticomuscular coherence (CMC) within the beta band (12–30 Hz). (Left) CMC laterality. Markers represent pre- and post-intervention laterality changes (more contralesional or more ipsilesional) during the extension and flexion tasks. * indicates a significant difference of *p* < 0.05. (Right) Correlation between changes in ipsilesional peak CMC and FMA during extension (*r* = 0.28, *p* = 0.472) and flexion (*r* = 0.76, *p* = 0.018).

Participants showed more involvement of the contralesional hemisphere after training during wrist extension but not during flexion. As shown in Figure 4, there was a significant change of beta band CMC laterality from the ipsilesional hemisphere toward the contralesional hemisphere during wrist extension (*t* = −2.70, *p* = 0.027). However, the laterality change in beta CMC during flexion was more widespread and not significant (*t* = 0.07, *p* = 0.944). Additionally, ipsilesional peak coherence within the beta band was significantly correlated with FMA during flexion (*r* = 0.76, *p* = 0.018) but not during extension (*r* = 0.28, *p* = 0.472).

### 3.4 Behavioral changes in clinical assessments

**Figure 5:**
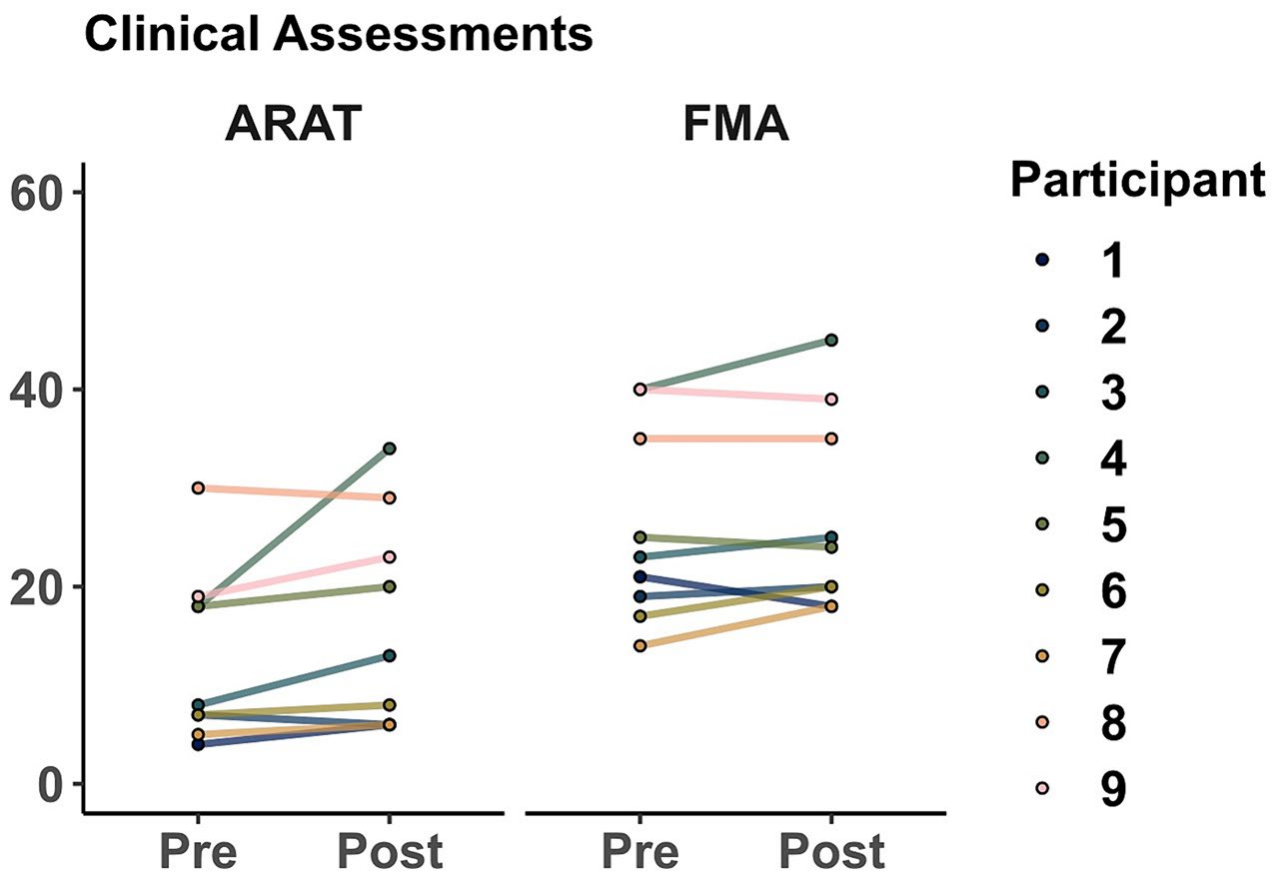
Clinical assessments before and after training. Markers represent the score for each participant as evaluated in sessions 1 (Pre) and 32 (Post). Action Research Arm Test (ARAT), Fugl-Meyer Assessment of the upper extremity (FMA).

As a group (shown in Figure 5), averaged ARAT increased from 12.88 ± 8.78 points to 16.11 ± 10.79 after training. However, this change was not significant (*t* = 1.86, *p* = 0.099). Similarly, averaged FMA increased from 26 ± 9.89 points to 27.11 ± 10. However, this change was not significant (*t* = 1.27, *p* = 0.238).

### 3.5 Training adherence and experience feedback

Participants demonstrated high adherence to the training protocol (mean = 85.5% ± 10%, of the 30 scheduled sessions), and considered that having hybrid sessions (e.g., with the research team monitoring two sessions each week via videoconferencing) was adequate to complete independent training for the remaining sessions. During the first week, a member of the research team monitored every session to facilitate successful use (in addition to an in-person orientation during the baseline clinical evaluations). Once hybrid sessions started (e.g., during the second week), setup time took approximately 10 min, including sensor placement and calibration. Overall, participants were eager to use Tele-REINVENT and shared that they would continue using it if given the opportunity, as it was a *fun* and *enjoyable experience*, that provided a *good and challenging workout*, and increased their agency in their recovery process. One participant stated that the training challenged use of “muscles that have been hibernating.” Furthermore, participants reported high levels of enjoyment (mean = 8.19 ± 1.33, out of 10 points) and rated the system as *working adequately* (mean = 7.80 ± 1.46, out of 10 points) in the daily experience questionnaire. Additionally, participants described positive changes in their overall quality of life, including, better sleep quality and increased awareness, sensation, flexibility, relaxation, and use of their impaired arm. Further descriptions and analyses regarding the feasibility and acceptability of this training program can be found in Donnelly et al. (2024).

## 4 Discussion

We conducted a pilot study of a portable EMG biofeedback system (Tele-REINVENT) for stroke survivors with moderate to severe upper extremity hemiparesis (*n* = 9). The system trains individuated muscle activation of wrist flexors and extensors using computer games that respond to real-time muscle activity. Tele-REINVENT was developed to enable high repetitions of isolated wrist extension and flexion to disrupt dysfunctional synergy patterns and improve motor control, particularly for those in the chronic phase of stroke for whom there is a lack of effective interventions. The portability of the system allows clients to use it as part of a home exercise program under the guidance of a rehabilitation therapist without hands-on assistance.

Analysis of our pilot data suggests that 30 sessions of remote training induced modest changes in clinical and physiological assessments. Additionally, changes in muscle individuation were correlated with a reduction in the perceived impact of stroke on participation and impairment of the affected side. Finally, the subjective experiences of participants provide evidence that Tele-REINVENT yielded noticeable changes in overall arm function and participation in their daily lives and would be a welcome addition to their rehabilitation. Based on our findings, we propose that Tele-REINVENT training can induce modest but important improvements to stroke survivors’ volitional movement, preparing them for other therapies that require higher baseline volitional movement, and improving their overall quality of life.

### 4.1 Quantitative improvements in motor control

Impaired arm motor control post-stroke affects performance in activities of daily living, and active range of motion is a good clinical predictor of upper extremity recovery (Beebe and Lang, 2008). We observed significant improvements in active wrist extension and flexion ROM, which were the target training movements. To examine more subtle changes to motor control, we also quantified the unique involvement of both wrist flexor and extensor muscle groups during EMG amplitude tracking tasks before and after training. In line with our previous work (Marin-Pardo et al., 2020, 2022), we observed a wide variation in the post-training improvements and non-significant group-level trends in the expected directions during both the wrist extension task (more activity from extensors than flexors) and the wrist flexion task (more activity from flexors than extensors). Inter-participant variation is expected in stroke rehabilitation outcomes given that there are many factors–socio-demographic, clinical, and genetic–that influence post-stroke motor recovery (Alawieh et al., 2018). Furthermore, people with more severe impairment in the acute phase show wider outcome variation in the chronic phase (Prabhakaran et al., 2008). Variation in treatment response even among subpopulations of stroke survivors, as seen in our data, has led to a shift in the field toward precision rehabilitation, which aims to “deliver the right intervention at the right time, to the right individual” (French et al., 2022). However, there are few known ‘right interventions’ for stroke survivors with chronic, severe sensorimotor impairment.

Many current evidence-based interventions for improving post-stroke sensorimotor outcomes require at least some volitional movement and have been primarily studied in people with mild to moderate impairment. For example, CIMT has been shown to be most effective for individuals with at least 20 degrees of baseline active wrist extension (“higher-functioning group”) and effective, albeit to a lesser extent, for those with at least 10 degrees of baseline active wrist extension (“lower-functioning group”) (Winstein et al., 2003; Wolf et al., 2008, 2010). In our sample, we observed a group-level improvement in wrist extension from a pre-training mean of 8.6 degrees to a post-training mean of 20.1 degrees (as seen in Table 2). At the individual level, we observed improvements that moved participants across the eligibility thresholds for CIMT (marked with dotted lines in Figure 2), advancing one participant into the “lower functioning group” and three participants into the “higher functioning group.” The participant who started in the “lower-functioning group” (Participant 5) had the greatest improvement in wrist extension and the second highest increase in Participation. Similarly, the Participants 2, 7, and 9, who advanced to the “higher-functioning group” were also among the highest improvements in ADL, FMA, and ARAT (respectively). Interestingly, Participant 8, who remained in the “lower-functioning group,” had the greatest improvement in active wrist flexion. These results, paired with their ER individuation changes, suggest that this participant might have relied more on their flexor muscles for both movement tasks. Notably, only the participant who started in the “higher-functioning group” (Participant 4) had improvements beyond established minimum clinically important differences in ARAT [16 of 5.7 points (Van der Lee et al., 2001)] and FMA [5 of 4.25 points (Page et al., 2012)]. Furthermore, Participants 6 and 7 were close to the FMA MCID cutoff (at 3 and 4 points, respectively) and, similarly, Participants 9 and 3 were close to the ARAT MCID cutoff (at 4 and 5 points, respectively). In addition, Participants 2 and 5 had the highest improvements in extension ROM and Participant 8 in flexion ROM. Therefore, we hypothesize that a minimum of 5 points of baseline ARAT might be necessary for Tele-REINVENT to induce functional improvements, as Participant 1 was the only who did not show improvements across FMA, ARAT, or ROM and had a baseline ARAT of 4 points. These findings suggest that training with Tele-REINVENT may help severe stroke survivors advance their volitional movement to the point of being eligible for other therapies for which they were previously ineligible, such as CIMT. This could also be true for other promising interventions used to treat mild to moderate impairment, requiring some functional and volitional movement. Some examples include robot-assisted paradigms (Mehrholz et al., 2018), neuromuscular and functional electrical stimulation-facilitated task-specific training interventions (Takeda et al., 2017), and virtual reality and game-based interventions with handheld controllers or movement tracking (Rand et al., 2014; Ikbali Afsar et al., 2018). Therefore, Tele-REINVENT presents an opportunity to improve sensorimotor outcomes for severe chronic stroke survivors with residual muscle activity and could be a precursor to other evidence-based interventions that require more volitional movement.

### 4.2 Quantitative motor control improvements are associated with subjective quality of life improvements

Despite non-significant group-level improvements in muscle individuation, we found a positive correlation between the improvement in ER during wrist extension and two SIS subscales after training: Participation and Impairment. Importantly, these associations were not present before the intervention. It is possible that this enhanced correlation post-treatment may provide a mechanistic explanation for how Tele-REINVENT may improve participation – that is, greater extensor control may have given participants more confidence in engaging in daily activities across diverse settings. Further research aimed at examining the relationship between physical recovery and participation is warranted. Additionally, participants qualitatively described modest improvement in their overall quality of life, including increased awareness, sensation, flexibility, relaxation, and use of their impaired arm. Previously, we reported qualitative findings about the acceptability of Tele-REINVENT, showing that participants perceived increased awareness of their residual arm movement and felt motivated to use it for everyday activities outside of the training paradigm, which contrasted with longstanding habits of compensating with the unaffected arm (Donnelly et al., 2023). Similarly, Wright and colleagues reported subjective improvements in hand function during activities of daily living after using EMG-biofeedback despite non-significant reduction in sensorimotor impairment (Wright et al., 2014). Additionally, Chen and colleagues found that stroke survivors experienced functional benefits beyond the intended physical benefits of a telerehabilitation intervention (Chen et al., 2020). Based on our previous findings, the findings of other groups, and the pilot data reported here, we theorize that through training with Tele-REINVENT, participants learned strategies (implicitly or explicitly) to isolate wrist movements and were able to transfer these strategies to their everyday activities, resulting in subjective improvements in participation and strength alongside modest improvements in objective measures. Specifically, though the training provided by Tele-REINVENT focused on individuation of wrist flexors and extensors, we also observed improvements in FMA and ARAT items that require isolated shoulder movement or coordination of the whole arm. Proximal changes in the upper extremity (i.e., shoulder, elbow) induced by distal training (i.e., wrist, hand) were also observed by Song et al. (2013) and Hu et al. (2015) among participants with moderate post-stroke arm impairment after robot-assisted training programs. Our combined results suggest that Tele-REINVENT training for the hand and wrist may yield widespread downstream recovery effects (e.g., reduced impact of stroke on daily activities, increased use of the affected arm, improved proximal arm motor control) beyond the ability to isolate a specific muscle group.

### 4.3 Neural reorganization characterized via corticomuscular coherence

Examining CMC among stroke survivors can be a useful method for determining which cortical regions may directly influence muscles of the affected arm, providing additional information regarding neural reorganization and stroke recovery. For example, CMC in the beta and gamma bands have been reported to be lower in moderate and severe chronic stroke than in neurotypical populations during arm reaching tasks (Fang et al., 2009). Furthermore, changes within these frequency bands have been suggested as an indicator of post-stroke recovery (von Carlowitz-Ghori et al., 2014; Liu et al., 2019). Similarly, CMC in the beta band may indicate a return to neurotypical patterns of brain activity (e.g., increasing or decreasing depending on the muscle and the task) in participants that show improved recovery (Chen et al., 2018).

At the group level, we observed a laterality change in beta band CMC (a shift from the ipsilesional to the contralesional hemisphere) during wrist extension movements after training. We also observed a positive correlation between FMA and peak CMC in the beta band during flexion movements. These changes may suggest reorganization of neural circuits supporting stroke recovery. For example, Divekar and John showed that peak beta CMC over the contralateral cortex was higher during wrist extension than in flexion in neurotypical participants (Divekar and John, 2013); our results may suggest an altered pattern of activity after stroke that might represent attempts for compensation during specific movements. These results might also be explained by other muscle activity patterns, e.g., flexion synergies that are typically associated with movement dysfunction after stroke (Ellis et al., 2017) or contributions from different neural sources (e.g., the reticulospinal drive) that may be larger for wrist flexors than extensors in neurotypical participants (Smith et al., 2019). Moreover, previous research has shown that participants with moderate to severe chronic stroke had higher CMC in the contralesional hemisphere for proximal muscles during finger movements (Guo et al., 2020). However, Wilkins and colleagues showed a shift from the contralesional to the ipsilesional hemisphere after using functional electrical stimulation therapy in moderate and severe chronic stroke (Wilkins et al., 2017). Dodd and colleagues found positive correlations of stroke recovery with increased contralesional and ipsilesional brain activity (Dodd et al., 2017). Finally, previous research suggest that higher peak CMC coherence is correlated with improved motor impairment (Krauth et al., 2019). However, further research with greater samples and accounting for possible covariates (e.g., chronicity, lesion location, lesion size) is necessary to better understand the possible mechanisms that might explain activity changes across the whole brain and their correlations with behavioral improvements after stroke.

### 4.4 Limitations and future directions

This pilot work shows that it is feasible to induce changes in clinical, physiological, and subjective outcome measures among chronic stroke survivors with moderate to severe hemiparesis via at-home training with Tele-REINVENT. However, due to the small sample size and relatively narrow inclusion criteria of our pilot study, analyzing the training effects of Tele-REINVENT among a more diverse sample was not within the scope of this paper. In future work, we plan to recruit stroke survivors with a greater diversity of time after stroke, level of impairment, and other socio-demographic and clinical factors to better understand how these and other covariates may affect the efficacy of the intervention.

### 4.5 Implications

Tele-REINVENT is a promising intervention that addresses current gaps in stroke rehabilitation. First, our pilot data show that training with Tele-REINVENT can induce observable changes in both clinical and physiological outcomes for chronic stroke survivors with severe impairment, a subpopulation that is currently underserved and experiences poor outcomes. Leveraging surface EMG technology, Tele-REINVENT enables measurement of small, incremental changes in muscle individuation and motor control. In a clinical setting, such data may be vital for reporting clinical progress and justifying the need for ongoing therapy services even in later stages of recovery. Second, the modest changes in clinical and physiological outcome measures observed in this study were associated with meaningful subjective improvements in strength, participation, and arm use in daily life. Finally, Tele-REINVENT may support recovery by reorganizing neural circuits, as evidenced by the laterality change in beta band CMC. Together, our findings suggest that Tele-REINVENT may be an effective intervention to stimulate motor control progress among those with chronic, severe hemiparesis and be a predecessor to other interventions for this subpopulation of stroke survivors that currently has limited access and eligibility for therapy.

## Data availability statement

The raw data supporting the conclusions of this article will be made available by the authors, without undue reservation.

## Ethics statement

The studies involving humans were approved by Institutional Review Board of the University of Southern California. The studies were conducted in accordance with the local legislation and institutional requirements. The participants provided their written informed consent to participate in this study.

## Author contributions

OM-P: Data curation, Formal analysis, Investigation, Methodology, Software, Visualization, Writing – original draft, Writing – review & editing. MD: Data curation, Formal analysis, Investigation, Methodology, Writing – original draft, Writing – review & editing. CP: Investigation, Software, Writing – review & editing. KW: Investigation, Writing – review & editing. S-LL: Conceptualization, Formal analysis, Funding acquisition, Methodology, Project administration, Resources, Supervision, Validation, Writing – review & editing.
